# Impact of Gender, Co-Morbidity and Social Factors on Labour Market Affiliation after First Admission for Acute Coronary Syndrome. A Cohort Study of Danish Patients 2001–2009

**DOI:** 10.1371/journal.pone.0086758

**Published:** 2014-01-30

**Authors:** Merete Osler, Solvej Mårtensson, Eva Prescott, Kathrine Carlsen

**Affiliations:** 1 Research Center for Prevention and Health, Glostrup Hospital, Glostrup, Denmark; 2 Department of Cardiology Y, Bispebjerg Hospital, Copenhagen, Denmark; 3 Institute of Public Health, University of Copenhagen, Copenhagen, Denmark; Iran University of Medical Sciences, Iran (Islamic Republic of)

## Abstract

**Background:**

Over the last decades survival after acute coronary syndrome (ACS) has improved, leading to an increasing number of patients returning to work, but little is known about factors that may influence their labour market affiliation. This study examines the impact of gender, co-morbidity and socio-economic position on subsequent labour market affiliation and transition between various social services in patients admitted for the first time with ACS.

**Methods:**

From 2001 to 2009 all first-time hospitalisations for ACS were identified in the Danish National Patient Registry (n = 79,714). For this population, data on sick leave, unemployment and retirement were obtained from an administrative register covering all citizens. The 21,926 patients, aged 18–63 years, who had survived 30 days and were part of the workforce at the time of diagnosis were included in the analyses where subsequent transition between the above labour market states was examined using Kaplan-Meier estimates and Cox proportional hazards models.

**Findings:**

A total of 37% of patients were in work 30 days after first ACS diagnosis, while 55% were on sick leave and 8% were unemployed. Seventy-nine per cent returned to work once during follow-up. This probability was highest among males, those below 50 years, living with a partner, the highest educated, with higher occupations, having specific events (NSTEMI, and percutaneous coronary intervention) and with no co-morbidity. During five years follow-up, 43% retired due to disability or voluntary early pension. Female gender, low education, basic occupation, co-morbidity and having a severer event (invasive procedures) and receiving sickness benefits or being unemployed 30 days after admission were associated with increased probability of early retirement.

**Conclusion:**

About half of patients with first-time ACS stay in or return to work shortly after the event. Women, the socially disadvantaged, those with presumed severer events and co-morbidity have lower rates of return.

## Introduction

Coronary heart diseases are among the leading causes of morbidity in Western societies. However, during recent years the prognosis has improved considerably, possibly due to the introduction of new invasive and medical treatments [1]. This has led to an increasing number of survivors returning to work after treatment. Established treatments such as percutaneous coronary interventions (PCI) and coronary artery bypass grafting (CABG) as well as patient factors such as gender, age, socio-economic position (SEP) and co-morbidity have been associated with the prognosis of acute coronary syndrome (ACS) [1], [2]. These factors also seem to influence the patient's transition from being a patient to returning to normality as maintaining an affiliation to the labour market.

Thus, two reviews have reported that 50–85% of patients in work with myocardial infarction maintain labour market affiliation but this proportion seems to depend on the size of the infarction, and the patient's age and education [3], [4]. Similar associations have been found for patients who have had a CABG [3]–[7] or PCI [7]. These studies have in most cases included small, selected patient groups and incomplete follow-up, particularly because outcome data in many studies were collected by questionnaire. In addition, few had examined the influence of gender and co-morbidity on labour market affiliation after ACS or coronary revascularisation.

One recent study, based on all 63,876 patients aged 30–63 years, who underwent a first CABG or PCI in Sweden between 1994 and 2006, examined sick leave after invasive treatments. This study showed that sick leave following coronary revascularisation was common, especially among women, and was also associated with SEP and co-morbidity [7]. In another population-based study from Finland, return to work was examined by the linkage of the Finnish cardiovascular register with a register for social security benefits for 5,074 patients aged 35–59 who had survived their first myocardial infarction (MI) between 1991 and 1996. This study showed that 58% of men and 56% of women were retained in work two years after their MI, but the authors did not examine whether any other patient or health factors influenced the probability of return to work [8].

Return to work after hospitalisation is in general dependent on gender, concomitant diseases, in particular diabetes, and socio-economic factors. A strong predictor for returning to work after hospitalisation is the occupational status at time of diagnosis. Aside from the recent study from Sweden [7], in the majority of studies conducted so far return to work after ACS, CABG or PCI is only considered for those persons who are in work at the time of diagnosis and thus represents a selected (and probably more healthy) part of the population included in the labour force. Further, in the majority of studies, the outcome has been dichotomised to working/not working. This is, however, a simplification of the occupational consequences of ACS, as in most welfare states not working can be split into different more or less voluntary schemes. Thus, knowledge is lacking on the impact of gender, co-morbidities and SEP on subsequent labour market affiliation and transition between various social services after discharge from hospital in an unselected group of patients surviving their first ACS event.

The aim of this study was therefore to examine the impact of patient's gender, co-morbidity and SEP on subsequent labour market affiliation (in work, unemployed, sick leave and disability or voluntary early retirement) in a cohort of all-Danish patients of working age who had been admitted for the first time for ACS and survived for 30 days thereafter.

## Materials and Methods

### Ethics statement

The study was based on linking information from four Danish population-based registers: The National Patient Register (NPR), The Danish Prescription Register (DPR), the Integrated Database for Labour Market Research (IDA) and the Register-based Evaluation of Marginalisation (DREAM) [9]–[11]. The study has been evaluated and approved by the Danish Data Protection Agency [9].

### Study population

The study population was derived from the NPR, which provides full histories of diseases leading to hospital admission and outpatient visits since 1979 and 1995, respectively [9]. This information includes dates of admission and discharge and diagnoses coded according to ICD-10 [9]. For this study, all first-time hospitalisations of ACS from the 1 January 2001 to 31 December 2009 were identified (n = 79,714) by the following specific ICD10 codes: I20.0 Unstable angina pectoris; I21.0–I21.3 ST-elevation myocardial infarction (STEMI); 121.4 NSTEMI and I21.9 AMI – Unspecified. ACS diagnoses in the NPR have acceptable coverage and validity, except for unstable angina pectoris [12]. Consequently, this discharge diagnosis should be used with caution. As the outcome under study is labour market participation after ACS, subjects outside the working age of 18–64 years (n = 49,072) and subjects retired due to disability or voluntary early pension before diagnosis (n = 7,706) were excluded from the study. As the majority of fatal events and post-operative complications occur within the first 30 days after diagnosis, we excluded subjects who died within the first 30 days after diagnosis (n = 1,010). This leads to a study population of 21,926 persons, aged 18–64 years, who were part of the workforce at the time of diagnosis and had survived 30 days after diagnosis.

### Outcome variables

The Danish labour market is characterised by a system with a high degree of economic compensation in the case of unemployment or reduced work ability, but also with a high turnover rate. Unemployed persons are warranted economic compensation if they are actively seeking work. During the study period, it was possible to receive a maximum of four years of unemployment benefit. At the end of these four years, or if a person was not qualified for unemployment benefit (i.e. not a member of a union) it was possible to receive social income. If a person was unable to work due to illness or disability, it was possible to receive sickness benefit for a maximum of 52 weeks during a period of two years or to apply for early retirement if the work ability was reduced to a level where it was not possible to hold down a job. This applies to all Danish citizens, independent of job type and insurance status. During the study period the retirement age was 64 years of age.

Transfer payments were obtained from DREAM, covering all citizens in Denmark who have received transfer payments from the state in any given episode since week 32 in 1991 [11]. The register was updated weekly until week 13 in 2011, providing between 65 and up to 568 weeks of follow-up. In this study, ‘in work’ was defined as not receiving any transfer payments. Transfer income was divided into sickness benefit, unemployment benefit (including social income) and permanent withdrawal from the workforce due to disablement or voluntary early retirement between the age of 60 and 64.

The main outcomes of the study were: 1. permanent withdrawal from the workforce before the age of 64 and thus receiving disability or voluntary early pension; 2. return to work among those who received sickness benefits or were unemployed 30 days after admission for ACS.

### Other co-variables

The following explanatory co-morbidity variables were obtained. From the NPR we acquired information on the specific ICD-10 ACS diagnosis as well as date and type of any invasive procedures, coronary angiography (CAG), percutaneous coronary interventions (PCI) and CABG. These variables were used as proxy measures for the severity of the cardiac event. Further information on co-morbidity five years preceding the year of diagnosis was drawn from NPR and DPR. The following diseases were included, compiled and dichotomised to yes/no: chronic obstructive pulmonary disease (COPD), asthma, cancer, diabetes and liver-, kidney-, connective-tissue or psychiatric diseases. The total number of co-morbidities was counted and grouped as 0; 1 to 2, and 3 or more.

Information on SEP was obtained from the IDA, which has been administrated by Statistics Denmark since 1980. The core variables in the database are derived once a year by linkage with Danish administrative registers [10]. Education was computed as the highest level of education registered in IDA and grouped in three categories: basic education (7–9 grade of obligatory schooling); medium education (high-school degree or vocational); higher education (more than high-school degree). Occupation was represented by a variable which specifies the character of employment during the year before diagnosis. The variable was categorised in three job types: at high level (in jobs requiring higher skills, including managers and self-employed with one or more employees), wage earners at basic level (in jobs requiring basic skills) and other (consisting of unemployed, under education or unspecified). Cohabitation status was categorised as single or living with a partner.

### Statistical analyses

We used Kaplan-Meier estimation and Cox proportional hazard regression analyses to estimate rate and rate ratios for the two main outcomes early retirement or return to work. Because age is a very strong predictor for the outcomes under study we used age as the underlying time variable. Person-years of follow-up were accumulated from age at 30 days after diagnosis and follow-up was terminated at the age of the event (retirement/return to work), death or censoring (age 64 or the end of follow-up – the last week of March 2011), whichever came first. We conducted multivariable analyses to evaluate the mutually adjusted effects of each of the covariables (gender, cohabitation status, education, occupation, ACS diagnosis, year of diagnosis, invasive treatment, co-morbidity and work status) on outcomes [13]. We also repeated the analyses in strata for each gender and sub-diagnosis, which did not indicate any specific interactions. Using Schoenfeld residuals and visual inspection of survival curves, we determined that estimated hazard ratios were constant over the follow-up time. All statistical analyses were carried out in STATA version 12.

## Results

### Baseline labour market affiliation and transitions

Of the 21,926 included patients, a total of 37% were in work 30 days after first-time admission with an ACS diagnosis, while 55% were on sick leave and 8% were unemployed (Table 1). Table 1 also shows that the probability of being in work was highest among male patients and those who were young, highest educated, living with a partner, had the most uncertain and possibly least severe events (diagnosed with unstable angina, had received no invasive treatment), were diagnosed 2007–2009, had no co-morbidities and were in work before diagnosis.

**Table 1:** Percentage in work, on sick leave and unemployed among patients, aged 18–64 years, admitted first time with acute coronary syndrome, who were part of the workforce at time of diagnosis and survived 30 days thereafter, in relation to gender, co-morbidity and socio-economic factors.

	In work	On sick leave	Unemployed	Chi-square test:p-value*
All (n = 21,926)	37.2	54.9	8.0	
*Gender*				
Men (n = 16739)	37.0	55.8	7.3	
Women (n = 5187)	38.0	51.7	10.2	p<0.01
Age				
18–29 (n = 344)	54.8	24.1	21.1	
30–39 (n = 2094)	42.9	42.2	14.9	p<0.01
40–49 (n = 6551)	36.3	53.7	10.0	p<0.01
50–59 (n = 11,010)	34.7	59.7	5.8	p<0.01
60–63 (n = 1927)	44.4	51.9	3.7	p<0.01
*Cohabitation*				
Living with a partner (n = 14,744)	38.1	56.4	5.5	
Single (n = 7011)	34.2	52.6	13.2	p<0.01
Education				
Basic (n = 6392)	30.4	57.9	11.8	
Medium (n = 10,071)	36.0	57.8	6.2	p<0.01
Higher (n = 4734)	47.5	47.5	5.0	p<0.01
*Occupation*				
Other (n = 2882)	28.6	27.8	43.4	
Wage earners at basic level (n = 12,927)	34.2	62.7	3.1	p<0.01
Wage earners at high level (n = 5997)	46.7	52.3	1.3	p<0.01
*ACS diagnosis*				
Unstable angina (n = 5475)	55.1	34.4	10.5	
AMI-STEMI (n = 5773)	27.1	65.8	7.1	p<0.01
NSTEMI (n = 4136)	30.5	63.2	6.3	p<0.01
AMI unspecified (n = 6542)	35.3	57.0	7.7	p<0.01
*Year of diagnosis*				
2001–2003 (n = 7738)	36.3	55.4	8.3	
2004–2006 (n = 7472)	36.0	55.2	8.8	p<0.01
2007–2009 (n = 6716)	39.5	53.2	6.6	p<0.01
*Procedures 30 days after admission*				
No invasive procedure (n = 5913)	54.0	34.4	11.7	
CAG, only (n = 8777)	33.0	60.0	7.0	p<0.01
PCI (n = 6257)	30.0	63.6	6.2	p<0.01
CABG (n = 802)	12.1	83.3	4.7	p<0.01
*Number of co-morbidities*				
None (n = 16969)	38.5	54.8	6.8	
1–2 (n = 4256)	31.5	56.4	12.2	p<0.01
3 and more (n = 701)	31.4	51.1	17.5	p<0.01
*Work status at time of diagnosis*				
In work (n = 11,150)	61.0	38.1	1.0	
Sick leave (n = 8231)	10.0	89.2	0.7	p<0.01
Unemployed (n = 2066)	3.9	21.3	75.8	p<0.01

* Marked category versus first variable category.

**Figure 1:**
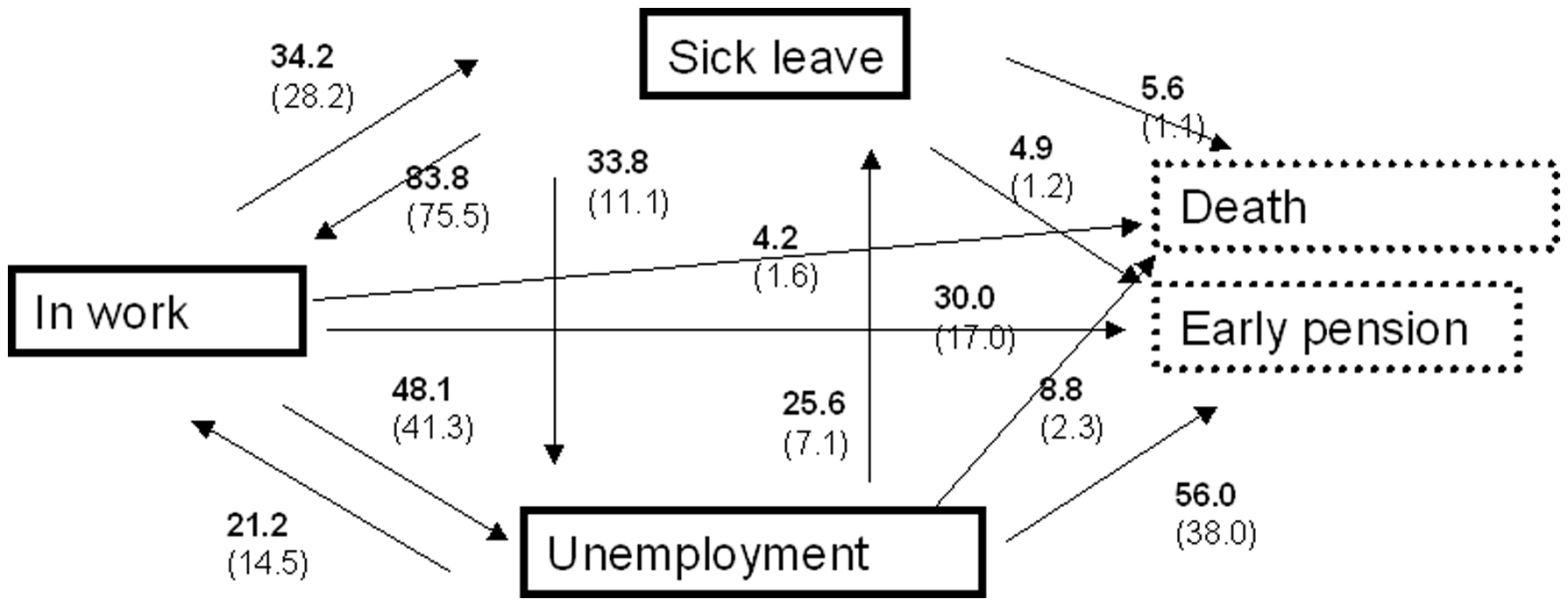
Patients with acute coronary syndrome's transition between various social services. Work, sick leave and unemployment cover persons in the workforce (3 left boxes with bold lines) at baseline for those aged 18–64 years. The lower box with dotted lines refers to early retirement independent of reason (disability or voluntary), which is an irreversible state, where persons are considered to leave the workforce forever (main outcome). Percentages in bold refer to how many patients ever experience the event during follow-up, while percentages in brackets refer to how many experience the transition as the first event after baseline.

As persons can change between the different states (in work, sick leave and unemployment) many times during follow-up, all the transitions between the different states are shown in Figure 1. Of those who were working during follow-up, 34% experienced one or more periods on sick leave and 30% were allocated to disability or voluntary early pension after one or more periods of sick leave and/or unemployment. The proportion in work increased, while the proportion on sick leave decreased during follow-up. Further, the proportion that was still a part of the workforce decreased. Thus, five years after ACS, 88% were still a part of the workforce. Among these, 65% were in work, 19% were unemployed and 16% were on sick leave (Table 2). The majority of patients who had left the labour force were under the age of 64 years and therefore receiving disability or voluntary early pension.

**Table 2:** Workforce participation 30 days, 1 year, 2 years and 5 years after acute coronary syndrome (ACS) in Denmark 2001–2009 among patients, aged 18–64 years, admitted first time with ACS, who were part of the workforce at time of diagnosis and survived 30 days thereafter (n = 21,926).

	30 days after time of diagnosis N (%)	1 year after diagnosis N (%)	2 years after diagnosis N (%)*	5 years after diagnosis N (%)*
**Part of the workforce**	21926 (100)	21869 (99.7)	19664(98.4)	15468 (88.0)
In work	8150 (37.2)	8777 (40.1)	8856 (45.0)	10.079 (65.2)
Unemployed	1743 (8.0)	1850 (8.5)	1936 (9.9)	2878 (18.6)
Sick leave	12033 (54.9)	11220 (51.4)	8852 (45.0)	2511 (16.2)
**Not part of the workforce**	0	35 (0.2)	249 (1.3)	1824 (10.4)
Disability or voluntary early pension	0 (0)	30 (85.7)	231 (92.8)	1751 (96.0)
Old-age pension	0 (0)	5 (14.3)	18 (7.2)	72 (4.0)
**Dead**	0 (0)	22 (0.1)	66 (0.3)	278 (1.6)

* Does not sum to total due to end of follow-up (n = 1947 after 2 years and n = 4356 after 5 years).

### Determinants of early retirement

**Figure 2:**
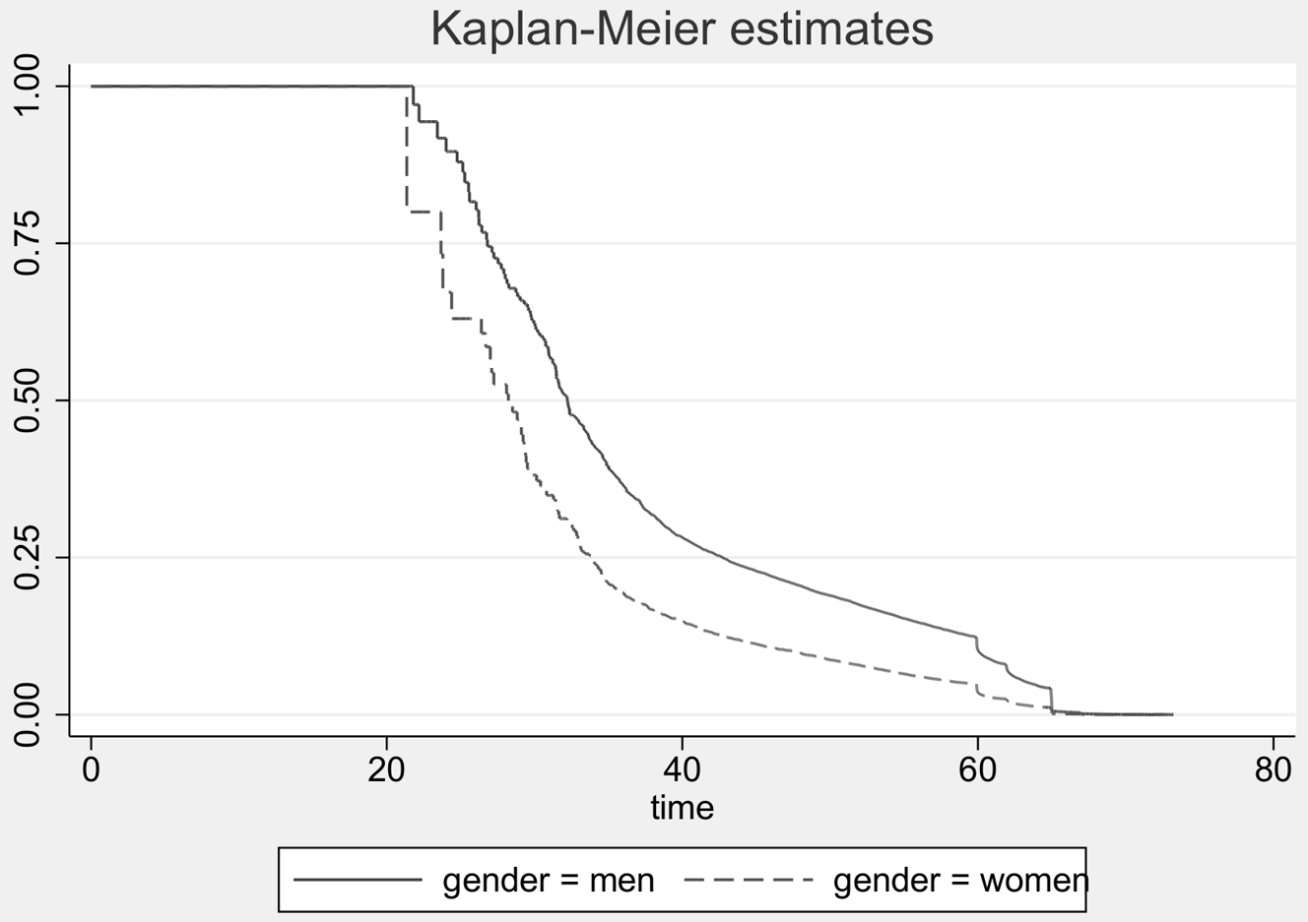
Kaplan-Meier estimates for early retirement after acute coronary syndrome by gender.

During follow-up a total of 43% withdrew permanently from the workforce. The mean number of years from 30 days after diagnosis to retirement, death or censoring was 4.1 years (median 3.7 years; range 0.0–10.2). Figure 2 shows the Kaplan-Meier estimates for men and women.

The probability of retirement due to disability or voluntary early pension was highest among women, patients above 50 years of age, those lowest educated, in basic or other jobs, living with a partner, who were on sick leave or unemployed 30 days after admission, who were diagnosed 2007–2009, had received CABG, and with co-morbidity (Table 3, first data column). The most prevalent co-morbidities were psychiatric disease (7.7%), diabetes (6.0%) and COPD (5.9%) and they were all associated with increased probability of early retirement – Hazard Ratio (HR) = 1.72(1.61–1.84), HR = 1.30(1.20–1.70) and 1.25(1.15–1.35), respectively (data not shown).

**Table 3:** The percentage and Hazard Ratio (HR) of disability or voluntary early pension after a mean follow-up of 4.1 years among patients, aged 18–64 years, admitted first time with acute coronary syndrome, who were part of the workforce at time of diagnosis and survived 30 days thereafter, in relation to gender, co-morbidity and socio-economic factors.

	% on disability or voluntary early pension	Crude*HR (95% CI)	Mutually adjusted HR (95% CI)
All (n = 21,926)	42.7		
*Gender*			
Men (n = 16739)	42.3	1	1
Women (n = 5187)	44.1	1.30(1.24–1.37)	1.30(1.24–1.37)
Age			
18–29 (n = 344)	39.2		
30–39 (n = 2094)	25.6		
40–49 (n = 6551)	19.9	*	*
50–59 (n = 11,010)	52.8		
60–63 (n = 1927)	81.5		
*Cohabitation*			
Living with a partner (n = 14,744)	44.4	1	1
Single (n = 7011)	38.8	1.08(1.04–1.13)	1.00(0.96–1.05)
*Education*			
Basic (n = 6392)	46.3	1	1
Medium (n = 10,071)	43.1	0.89(0.85–0.93)	0.97(0.96–1.03)
Higher (n = 4734)	37.8	0.68(0.64–0.72)	0.85(0.83–0.91)
*Occupation*			
Other (n = 2882)	52.5	1.97(1.84–2.10)	1.57(1.43–1.69)
Wage earners at basic level (n = 12,927)	43.6	1.46(1.39–1.54)	1.32(1.24–1.37)
Wage earners at high level (n = 5997)	36.7	1	1
*ACS diagnosis*			
Unstable angina (n = 5475)	40.2	1	1
AMI-STEMI (n = 5773)	42.8	1.12(1.06–1.20)	1.01(0.95–1.07)
NSTEMI (n = 4136)	42.7	1.10(1.07–1.17)	0.97(0.93–1.04)
AMI unspecified (n = 6542)	44.7	1.08(1.02–1.14)	1.00(0.94–1.04)
*Year of diagnosis*			
2001–2003 (n = 7738)	57.8	1	1
2004–2006 (n = 7472)	43.7	1.10 (1.05–1.15)	1.07(1.02–1.12)
2007–2009 (n = 6716)	24.1	1.20 (1.13–1.27)	1.18(1.12–1.26)
*Procedures 30 days after admission*			
No invasive procedure (n = 5913)	44.4	1	1
CAG, only (n = 8777)	40.6	1.14(1.08–1.20)	1.03(0.98–1.11)
PCI (n = 6257)	42.7	1.13(1.07–1.19)	1.05(0.99–1.13)
CABG (n = 802)	60.0	1.29(1.17–1.42)	1.08(0.75–1.20)
*Number of co-morbidities*			
None (n = 16969)	39.9	1	1
1–2 (n = 4256)	51.3	1.45 (1.38–1.58)	1.35 (1.28–1.42)
3 and more (n = 701)	58.5	1.62 (1.47–1.79)	1.51 (1.37–1.68)
*Work status at time of diagnosis*			
In work (n = 11,150)	30.4	1	1
Sick leave (n = 8231)	49.1	3.15(2.92–3.40)	1.90(1.81–2.01)
Unemployed (n = 2066)	56.0	1.98(1.89–2.07)	2.94(2.72–3.19)

* age underlying timescale.

When age at retirement was taken into account female gender, living single, low education, basic occupation, receiving sickness benefits or being unemployed 30 days after admission, co-morbidity, ACS-diagnosed 2007–2009, unspecific and greater severity of events (being diagnosed with STEMI, NSTEMI and unspecified AMI and receiving invasive treatment) were associated with increased rates of early retirement (Table 3, second data column). When all factors were mutually adjusted, the estimates for cohabitation status, the ACS sub-diagnosis, and treatment attenuated and became insignificant.

### Determinants of return to work

**Figure 3:**
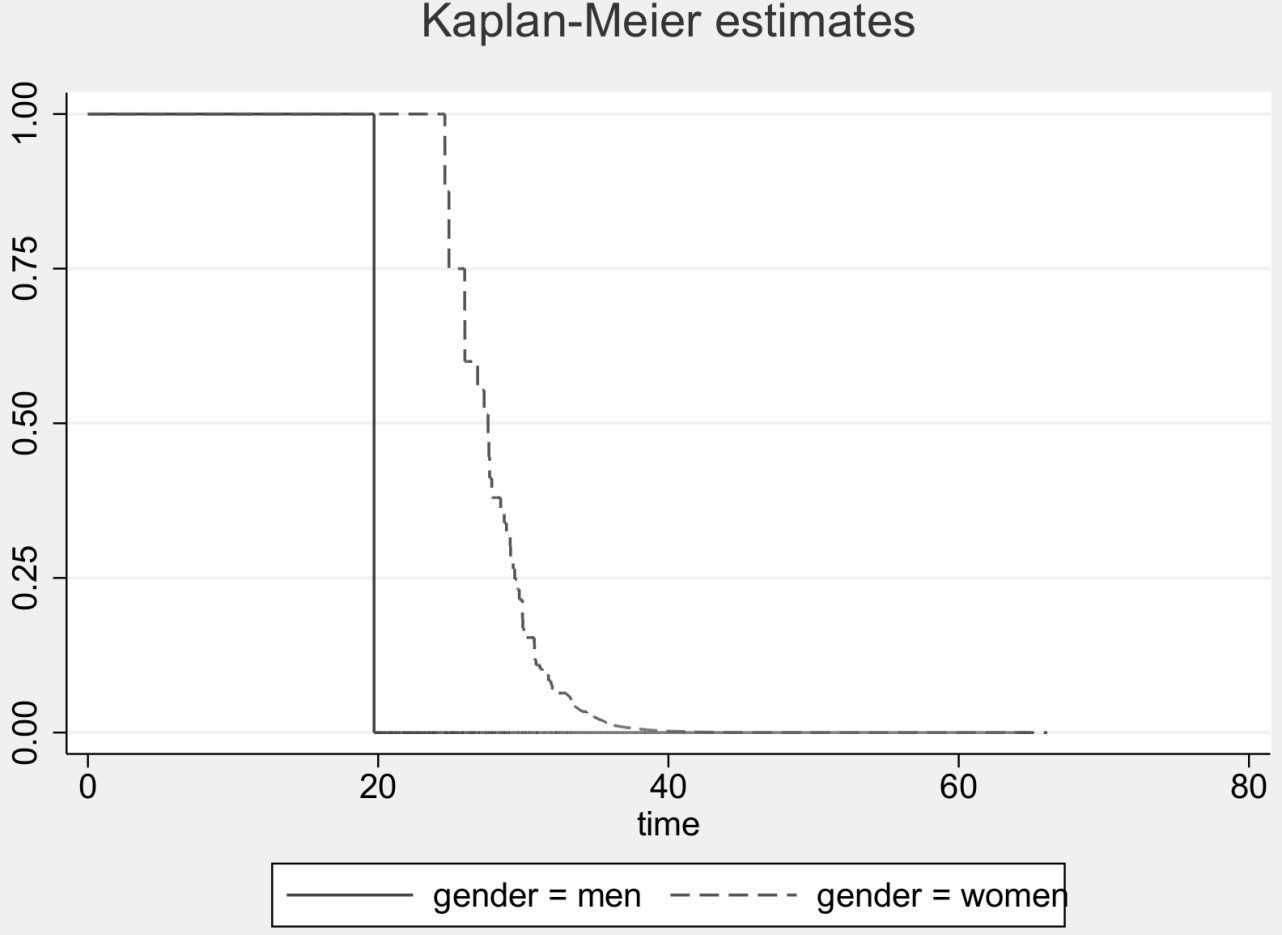
Kaplan-Meier estimates for return to work after acute coronary syndrome by gender.

Among the 13,776 patients who were unemployed or on sick leave 30 days after the index event, 79% returned to work at least once during follow-up. Mean years of follow-up was 1.3 (median 0.3 years; range 0.0–10.0). Figure 3 provides the Kaplan-Meier estimates for men and women. The probability of returning to work was highest among male patients, below 50 years of age, those living with a partner, highest educated, with higher occupations, being diagnosed 2002–2003, with less severe events (NSTEMI, having PCI), who were on sick leave 30 days after admission, and with no co-morbidity (Table 4). Patients with psychiatric disease, diabetes or COPD were less likely to return to work (HR = 0.39(0.37–0.41); 0.58(0.54–0.62); and 0.62(0.56–0.67), respectively). The same factors were associated with increased rates of return to work when age was taken into account (Table 4, second column). However, the HR for year of diagnosis were reversed. Similar was seen after controlling for potential confounders (Table 4, third column). Compared to the group of persons in work 30 days after diagnosis the mean number of years from inclusion to retirement or censoring was much lower (0.9 years) among unemployed or sick-listed ACS patients.

**Table 4:** The percentage and Hazard Ratio (HR) of return to work among patients, aged 18–64 years, admitted first time with acute coronary syndrome, who were on sick leave or were unemployed 30 days after diagnosis in relation to gender, co-morbidity and socio-economic factors.

	% return to work	Crude HR (95% CI*)	Mutually adjusted HR (95% CI)
All (n = 13776)	79.3		
*Gender*			
Men (n = 10561)	81.3	1	1
Women (n = 3217)	72.9	0.61(0.60–0.64)	0.66(0.63–0.70)
*Age*			
18–29 (n = 152)	80.1		
30–39 (n = 1195)	80.3		
40–49 (n = 4170)	81.5	*	*
50–59 (n = 7181)	78.3		
60–63 (n = 1080)	77.1		
*Cohabitation*		1	1
Living with a partner (n = 9132)	82.3	0.60(0.58–0.63)	0.72(0.70–0.76)
Single (n = 4616)	73.5		
*Education*			
Basic (n = 4454)	74.6	1	
Medium (n = 6442)	81.0	1.40(1.35–1.47)	1.13(1.08–1.13)
Higher (n = 2488)	85.5	1.85(1.77–1.97)	1.43(1.35–1.52)
*Occupation*			
Other (n = 2057)	45.8	0.08(0.07–0.09)	0.15(0.14–0.17)
Wage earners at basic level (n = 8507)	82.9	0.53(0.51–0.56)	0.68(0.60–0.71)
Wage earners at higher level (n = 3199)	91.5	1	
*ACS diagnose*			
Unstable angina (n = 2460)	75.1	1	1
AMI-STEMI (n = 4212)	80.1	1.42(1.36–1.51)	0.99(0.93–1.05)
NSTEMI (n = 2875)	81.7	1.64(1.54–1.74)	1.28(1.11–1.25)
AMI unspecified (n = 4231)	79.3	1.25(1.19–1.33)	0.98(0.92–1.04)
*Year of diagnose*			
2001–2003 (n = 4931)	80.8	1	1
2004–2006 (n = 4783)	78.5	1.19(1.15–1.25)	1.12(1.07–1.18)
2007–2009 (N = 4062)	77.1	1.80 (1.72–1.89)	1.51(1.44–1.59)
*Procedures 30 days after admission*			
No invasive procedure (n = 2724)	75.0	1	1
CAG, only (n = 5880)	79.6	1.56(1.49–1.64)	1.12(0.95–1.08)
PCI (n = 4363)	82.1	1.71(1.67–1-86	1.16 (1.11–1.22)
CABG (n = 708)	78.2	1.42(1.29–1.36)	0.89 (0.81–0.98)
*Number of comorbidities*			
None (n = 10,421)	83.7	1	1
1–2 (n = 2871)	67.9	0.48(0.45–-0.50)	0.50(0.48–0.53)
3 and more (n = 484)	51.4	0.28(0.25–0.32)	0.29(0.23–0.34)
*Work status 30 days after diagnose*			
Sick leave (n = 12029)	83.8	1	1
Unemployed (n = 1747)	48.1	0.16(0.15–0.18)	0.18(0.17–0.20)

* age underlying timescale.

## Discussion

This population-based cohort study showed that around 79% of patients of working age with a first-time ACS diagnose either continued working or returned to work once during a mean follow-up of 1.3 years

Most patients experienced some shifts between the mutually exclusive states work, sick leave and unemployment before they reached the irreversible state of early retirement. In total 43% patients left the labour market either due to disability or voluntarily. This proportion of patients who retired early seems to be in accordance with the findings in the Finnish register-based study where 40% of patients with MI aged 35–50 were on a disability pension two years after the event, while 62% and 57% were in work after one and two years, respectively [8]. In the present study, of all ACS patients the percentage in work after one and two years was somewhat lower – 40% and 45%, respectively.

We also found that the probability of return to work was highest in males, those with high SEP, no co-morbidity, with NSTEMI diagnosis and who had a PCI. Over the last decades, studies have shown that younger women (<70 years) have a poorer prognosis than their male peers [14], [15]. Our and the Swedish study on CABG and PCI patients [7] suggest that such a gender difference also applies to women's work affiliation.

Low SEP has predicted lower rates of return to work in most other studies [3]–[5], [7]. This might reflect that high SEP jobs are more flexible with regard to taking changes in work capacity into account and that work ability in low SEP jobs is more affected by ACS than in high SEP jobs. In the present study co-morbidity, in particular major psychiatric diseases (schizophrenia/depression), was prevalent and associated with a lower rate of return to the labour market. The previous studies from Finland and Sweden [5], [7] also found that co-morbidity was associated with labour market affiliation, and in both studies this was most evident for diabetes. However, the Finnish study had only included somatic diseases, while the Swedish comprised both somatic and psychiatric co-morbidity.

Fewer have examined the impact of ACS sub-diagnosis and invasive procedures on return to work, since most studies have been restricted to specific diagnosis (mainly MI) or procedures (mainly CABG) [16]–[18]. In a study from the 1990s Mark et al. found no significant one–year return-to-work rate among 1,252 ACS patients receiving initial percutaneous transluminal coronary angioplasty or CABG versus initial medical therapy [19]. Similarly, in two randomised controlled trials there was no difference in the number of persons returning to work after PCI and CABG [20], [21]. Our finding of a more favourable job prognosis for NSTEMI events and PCI-treated patients might reflect that these variables are proxies for the severity of the disease. Further, these patients might get through the event more easily due to more specific treatment regimes.

The number of patients allocated to disability or voluntary early pension differed between the three labour market groups. Those who experienced one or more episodes of unemployment had the highest risk of early retirement pension, while those in work had the lowest. This finding could be caused by the contextual legislation in Denmark where sickness benefit is only assigned for 52 weeks, after which time the person is supposed to return to work or take early retirement pension. The observed increased risk for pension after unemployment could then be caused by the fact that patients with ACS return to work after 52 weeks of sickness benefit and experience that the balance between job demands and perceived work ability is out of adjustment and then have to quit their job. Another explanation lies in the fact that the highest risk factor for unemployment is previous episodes of unemployment, and that unemployed persons in general are known to be less healthy than persons in work, which could point towards an increased risk for early retirement pension among ACS patients who were unemployed before diagnosis.

### Strengths and limitations

The primary strength of this study is the large patient population which covers all patients admitted first time with ACS in the period from 2001 to 2009 in Denmark. The patients were identified in the NPR and data from this register is considered to have a high quality for patients with a coronary heart disease diagnosis. Thus, a previous study found a positive predictive value for myocardial infarction in the NPR of around 90% [12], [22]. However, the positive predictive value was lower (around 45%) for unstable angina. The information on determinants in the present study is based on data from nationwide registers with high completeness and good validity and missing values are random and not associated with the outcome under study, whereby selection-bias is removed. Variables regarding socio-economic position and affiliation to the labour market are administrative data collected prospectively, why recall bias is eliminated. Our study also has, however, some limitations. First of all we were not able to include more detailed information on work environment, which has been shown to be associated with the possibilities of reductions in work hours and reassignment to other work tasks. We defined return to work as not receiving any transfer payments, which can lead to misclassification of persons leaving the workforce without receiving economic compensation from the state. This is, however, very rare in Denmark and can be ignored in this study. We used invasive procedures during the first 30 days after diagnosis as covariates in our study. Thus, around 10% of those who only had a CAG actually had a PCI later. This information was not included in the analyses but might be a factor that could have an impact on return to work. Since some patients die during follow-up there might be a problem with competing risk [23]. However, we did not use competing risk analysis, as the number of deaths was relatively small. Furthermore, such models do not provide simple relationships between variables [23].

The present study is conducted in a Nordic welfare system with high turnover rates in the labour market, high rates of participation and high degrees of social security. Expenditures for social protection in the Nordic countries including Denmark is relatively high compared to the rest of the European Union and countries such as the US and Canada, but these countries all have some degree of a social welfare system and universal health care. The size of economic compensation and duration of sick leave might have an impact on the consequence of a chronic disease but the risk factors and reasons for being on sick leave or returning to work is not only influenced by the political context but also by an individual's behaviour.

## Conclusion

In this study more than half of patients with first-time ACS stay in or return to work shortly after the event. Women, the socially disadvantaged, those with presumed more severe cardiac events and co-morbidity have lower rates of return when other clinical factors are accounted for. These factors should be considered during physical and social rehabilitation.
